# Multi-Modal Medical Image Fusion With Geometric Algebra Based Sparse Representation

**DOI:** 10.3389/fgene.2022.927222

**Published:** 2022-06-23

**Authors:** Yanping Li, Nian Fang, Haiquan Wang, Rui Wang

**Affiliations:** ^1^ School of Communication and Information Engineering, Shanghai University, Shanghai, China; ^2^ Office of Academic Affairs, Shanghai University, Shanghai, China; ^3^ Department of General Surgery, Shanghai General Hospital of Shanghai Jiaotong University, Shanghai, China

**Keywords:** multi-modal medical image, sparse representation, geometric algebra, image fusion, dictionary learning (DL)

## Abstract

Multi-modal medical image fusion can reduce information redundancy, increase the understandability of images and provide medical staff with more detailed pathological information. However, most of traditional methods usually treat the channels of multi-modal medical images as three independent grayscale images which ignore the correlation between the color channels and lead to color distortion, attenuation and other bad effects in the reconstructed image. In this paper, we propose a multi-modal medical image fusion algorithm with geometric algebra based sparse representation (GA-SR). Firstly, the multi-modal medical image is represented as a multi-vector, and the GA-SR model is introduced for multi-modal medical image fusion to avoid losing the correlation of channels. Secondly, the orthogonal matching pursuit algorithm based on geometric algebra (GAOMP) is introduced to obtain the sparse coefficient matrix. The K-means clustering singular value decomposition algorithm based on geometric algebra (K-GASVD) is introduced to obtain the geometric algebra dictionary, and update the sparse coefficient matrix and dictionary. Finally, we obtain the fused image by linear combination of the geometric algebra dictionary and the coefficient matrix. The experimental results demonstrate that the proposed algorithm outperforms existing methods in subjective and objective quality evaluation, and shows its effectiveness for multi-modal medical image fusion.

## 1 Introduction

Medical Image fusion technology integrates technologies in many fields such as computer technology, sensor technology, artificial intelligence, and image processing. It comprehensively extracts image information collected by different sensors and concentrates all the information of the image, which can reduce the information redundancy of the image, enhance the readability of the image and provide more specific disease information for diagnosis (Riboni and Murtas, 2019; Li et al., 2021; Wang et al., 2022).

According to the types of fused images, medical image fusion can be divided into unimodal medical image fusion and multi-modal medical image fusion (Tirupal et al., 2021). A unimodal medical image refers to multiple images of a patient’s organ collected by the same device, which are combined into one image by corresponding fusion algorithm. The purpose is to collect image information under different contrasts (Zhang. et al., 2021). Multi-modal medical images refer to images obtained by different imaging methods. Different types of medical images contain different information, and the obtained fused image can summarize various feature information to provide medical staff with more comprehensive pathological information (Zhu et al., 2017). Common medical images include CT images, MR images, and SPECT images (Thieme et al., 2012; Nazir et al., 2021; Engudar et al., 2022).

Multi-modal medical image fusion mainly includes the following methods: morphological methods, knowledge based methods, wavelet based methods, neural network based methods, methods based on fuzzy logic, and so on (James and Dasarathy, 2014). Naeem used discrete wavelet transform (DWT) to fuse images with different details, which changed the uniformity of the details contained in the fused image (Naeem et al., 2016). Guruprasad et al. (2013) proposed an image fusion algorithm based on DWT-DBSS and use the maximum selection rule to obtain detailed fusion coefficients. Bruno presents a novel Wavelet-based method to fuse medical images according to the MRA approach, that aims to put the right “semantic” content in the fused image by applying two different quality indexes: variance and modulus maxima (Alfano et al., 2007). A hierarchical image fusion scheme is presented which preserves the details of the input images of most relevance for visual perception (Marshall and Matsopoulos, 2002).

Sparse representation (Shao et al., 2020) can deal with the natural sparsity of signals by the physiological properties of the human visual system, which is a linear combination of dictionary atoms and sparse coefficients to represent the signal with as few atoms as possible in a given overcomplete dictionary. Bin Yang and Shutao Li (2010) first introduced sparse representation into image fusion, and adopted the sliding window technique to make the fusion process robust to noise and registration. Zong and Qiu (2017) proposed a fusion method based on classified image blocks, which used the directional gradient histogram feature to classify image blocks to establish a sub-dictionary. It can reduce the loss of image details and improve the quality of image fusion.

Traditional sparse representation fusion method usually processes the color channels separately, which easily destroys the correlation between image channels and results in loss of color in the fused image. Geometric algebra (GA) has been considered as one of the most powerful tools in multi-dimensional signal processing and has witnessed great success in a wide range of applications, such as physics, quantum computing, electromagnetism, satellite navigation, neural computing, camera geometry, image processing, robotics, and computer vision, et al. (da Rocha and Vaz, 2006; Wang et al., 2019a; Wang et al., 2021a). Inspired by the paper (Wang et al., 2019b), the geometric algebra-based sparse representation (GA-SR) is introduced for multi-modal medical image fusion in this paper.

The rests of this paper are organized as follows. In Section 2, this paper introduces the basic knowledge of geometric algebra. Section 3 introduces the GA-SR algorithm and the fusion steps of the proposed algorithm. Section 4 provides the experimental analysis including subjective and objective quality evaluations. Finally, Section 5 concludes the papers.

## 2 Geometric Algebra

Geometric algebra combines quaternions and Grassmann algebras, which can extend operations to higher-dimensional spaces. The geometric algebra space does not rely on coordinate information for calculation (Batard et al., 2009), and all geometric operators are included in the space. Any multi-modal medical image can be represented by geometric algebraic orthonormal base as a multi-vector for overall processing, which can ensure the correlation between each channel of the image (da Rocha and Vaz, 2006; López-González et al., 2016).

Geometric algebra is generated by quadratic space, and is defined as follows. Let $G_{n}$ denote the $2^{n}$ dimensional geometric algebraic space generated by the orthonormal basis vectors $\left\{\gamma_{1},\gamma_{2},\ldots ,\gamma_{n}\right\}$ , including the following complete orthonormal base: Eq. 1

$$\left\{1,\left\{\gamma_{i}\right\},\;\left\{\gamma_{i}\gamma_{j}\right\},\;\cdots \;,\left\{\gamma_{1}\gamma_{2}\cdots \gamma_{n}\right\}\right\}$$ (1)

For example, the orthonormal base of $G_{3}$ vector space consists of $2^{3}=8$ vectors, which are $\left\{1,\;\gamma_{1},\;\;\gamma_{2},\;\gamma_{3},\;\;\gamma_{12},\;\;\gamma_{13},\;\gamma_{23}\right\}\;$ .

The orthonormal base in the geometric algebraic space $G_{n}$ satisfies the following basic operation rules, Eqs 2–5

$$\gamma_{i}\wedge \gamma_{j}=-\gamma_{j}\wedge \gamma_{i},\;\;\;i\neq j,\;\;\;\;\;\;\;\;\;\;\;i,j=1,2,\ldots ,n$$ (2)

$$\;\gamma_{i}^{2}=1,\;\;\;\;\;\;\;\;\;\;\;\;\;\;\;\;\;\;\;i,j=1,2,\ldots ,n$$ (3)

$$\gamma_{i}\cdot \gamma_{j}=\frac{1}{2}\left(\gamma_{i}\gamma_{j}+\gamma_{j}\gamma_{i}\right),\;\;i,j=1,2,\ldots ,n$$ (4)

$$\gamma_{i}\gamma_{j}=\gamma_{i}\cdot \gamma_{j}+\gamma_{i}\wedge \gamma_{j},\;\;i,j=1,2,\ldots ,n$$ (5)

where ∧ represents the outer product symbol, represents the inner product symbol, $\gamma_{i}\gamma_{j}$ represents the geometric product of $\gamma_{i}$ and $\gamma_{j}$ , which is equal to the sum of the inner and outer products of $\gamma_{i}$ and $\gamma_{j}$ .

## 3 Geometric Algebra Based Sparse Representation Based Multi-Modal Medical Image Fusion Based on

In this section, the GA-SR based multi-modal medical image fusion is provided.

### 3.1 Geometric Algebra Based Sparse Representation Model

The sparse representation model of GA multi-vector can be defined as

$$min_{a}\|a{\|}_{0},\;\;s.t.q=Da$$ (6)

where $D=\left(E_{0}\left(D\right)+\underset{1\leq i\leq n}{\sum }E_{i}\left(D\right)\gamma_{i}+\underset{1\leq i<j\leq n}{\sum }E_{ij}\left(D\right)\gamma_{ij}+\cdots +E_{1\cdots n}\left(D\right)\gamma_{1\cdots n}\in {\left(G_{n}\right)}^{N\times M}\right)$ is a geometric algebra dictionary containing *M* dictionary atoms, and $a=\left(E_{0}\left(a\right)+\sum_{1\leq i\leq n}E_{i}\left(a\right)\gamma_{i}+\sum_{1\leq i<j\leq n}E_{ij}\left(a\right)\gamma_{ij}+\cdots +E_{1\cdots n}\left(a\right)\gamma_{1\cdots n}\in {\left(G_{n}\right)}^{M}\right)$ is a sparse coefficients vector in geometric algebra form. $\|a{\|}_{0}$ is the objective function, which is used to calculate the number of non-zero vectors in the vector *a*. The multi-modal medical image based on the GA-SR model can be described in the Eq. 7

$$\left[0\cdots E_{i}\left(q\right)\cdots E_{ij}\left(q\right)\cdots E_{1\cdots n}\left(q\right)\right]=\left[E_{0}\left(D\right)\cdots E_{i}\left(D\right)\cdots E_{ij}\left(D\right)\cdots E_{1\cdots n}\left(D\right)\right]\bar{a}$$ (7)

For a three-channel multi-modal medical image $Q^{\prime }\in {\left(G_{2}\right)}^{N\times K}$ , it is assumed that each image block $q^{\prime }\in {\left(G_{2}\right)}^{\sqrt{N}\times \sqrt{N}}$ can be converted into a vector $q^{\prime }\in {\left(G_{2}\right)}^{N}$ of length *N*, and the vector $q^{\prime }$ can be expressed as shown in the Eq. 8

$$q^{\prime }=0+E_{1}\left(q^{\prime }\right)\gamma_{1}+E_{2}\left(q^{\prime }\right)\gamma_{2}+E_{12}\left(q^{\prime }\right)\gamma_{12}\in {\left(G_{2}\right)}^{N}$$ (8)

For a three-channel multi-modal medical image, its sparse representation model can be defined as shown in the Eq. 9

$$min_{a^{\prime }}\|a^{\prime }{\|}_{0},\;\;s.t.q^{\prime }=D^{\prime }a^{\prime }$$ (9)

Where $D^{\prime }=E_{0}\left(D^{\prime }\right)+E_{1}\left(D^{\prime }\right)\gamma_{1}+E_{2}\left(D^{\prime }\right)\gamma_{2}+E_{12}\left(D^{\prime }\right)\gamma_{12}$ , $D^{\prime }\in {\left(G_{2}\right)}^{N\times M}$ is a three-channel geometric algebra dictionary consisting of *M* dictionary atoms, $a^{\prime }=\left(E_{0}\left(a^{\prime }\right)+E_{1}\left(a^{\prime }\right)\gamma_{1}+E_{2}\left(a^{\prime }\right)\gamma_{2}+E_{12}\left(a^{\prime }\right)\gamma_{12}\right)$ is the corresponding geometric algebra coefficient vector, and $\|a^{\prime }{\|}_{0}$ is used to calculate the number of non-zero elements in the vector $a^{\prime }$ .

Therefore, the GA-SR model of the three-channel medical image can be described as follows

$$\left[0\;\;E_{1}\left(q^{\prime }\right)\;\;E_{2}\left(q^{\prime }\right)\;\;E_{12}\left(q^{\prime }\right)\;\right]=\left[E_{0}\left(D^{\prime }\right)\;\;\;E_{1}\left(D^{\prime }\right)\;\;E_{2}\left(D^{\prime }\right)\;\;E_{12}\left(D^{\prime }\right)\right]a^{\prime }$$ (10)

The general form of a three-channel medical image sparse coefficient matrix can be obtained by

$$\left(a^{\prime }\right)=\left[\begin{array}{cccc} E_{0}\left(a^{\prime }\right) & E_{1}\left(a^{\prime }\right) & E_{2}\left(a^{\prime }\right) & -E_{12}\left(a^{\prime }\right)\\ E_{1}\left(a^{\prime }\right) & E_{0}\left(a^{\prime }\right) & -E_{12}\left(a^{\prime }\right) & E_{2}\left(a^{\prime }\right)\\ E_{2}\left(a^{\prime }\right) & E_{12}\left(a^{\prime }\right) & E_{0}\left(a^{\prime }\right) & -E_{1}\left(a^{\prime }\right)\\ E_{12}\left(a^{\prime }\right) & E_{2}\left(a^{\prime }\right) & -E_{1}\left(a^{\prime }\right) & E_{0}\left(a^{\prime }\right) \end{array}\right]$$ (11)

### 3.2 The Representation of Multi-Modal Medical Image

Any pixel *F* of a multi-modal medical image can be represented as a multi-vector form in $G_{n}$ space, as shown in the Eq. 12

$$\begin{array}{l} F\left(x,y\right)=F_{0}\left(i,j\right)+\underset{1\leq i\leq n}{\sum }F_{i}\left(i,j\right)\gamma_{i}+\underset{1\leq i<j\leq n}{\sum }F_{ij}\left(i,j\right)\gamma_{ij}+\cdots +\;F_{1\cdots n}\left(i,j\right)\gamma_{1\cdots n},\\ F\left(i,j\right)\in R \end{array}$$ (12)

While $\gamma_{i},\;\gamma_{ij},\;\gamma_{1\ldots n}$ are the orthonormal base of geometric algebra, and $F_{i}\left(i,j\right),\;F_{ij}\left(i,j\right),\cdots ,\;F_{1\cdots n}\left(i,j\right)$ represent the pixels of the multi-modal medical image at (i,j) . Each channel of a multi-modal medical image can be encoded on an orthonormal basis of geometric algebra. Therefore, a multi-modal medical image Q of size M×N and $Q\in {\left(G_{n}\right)}^{M\times N}$ can be expressed as

$$\begin{array}{l} Q=E_{0}\left(Q\right)+\underset{1\leq i\leq n}{\sum }E_{i}\left(Q\right)\gamma_{i}+\underset{1\leq i<j\leq n}{\sum }E_{ij}\left(Q\right)\gamma_{ij}+\cdots +E_{1\cdots n}\left(Q\right)\gamma_{1\cdots n}\;,\\ E\left(Q\right)\in {\left(R\right)}^{M\times N} \end{array}$$ (13)

Assuming that each image block of the multi-modal medical image $Q\in {\left(G_{n}\right)}^{N\times K}$ is $q\in {\left(G_{n}\right)}^{\sqrt{N}\times \sqrt{N}}$ , where *N* represents the size of image and *K* represents the number of image patches, which can be converted into a vector form of length *N*, and the geometric algebra form of the image block *q* is shown in the Eq. 14

$$\begin{array}{l} q=E_{0}\left(q\right)+\underset{1\leq i\leq n}{\sum }E_{i}\left(q\right)\gamma_{i}+\underset{1\leq i<j\leq n}{\sum }E_{ij}\left(q\right)\gamma_{ij}+\cdots +E_{1\cdots n}\left(q\right)\gamma_{1\cdots n},\\ E\left(q\right)\in R \end{array}$$ (14)

### 3.3 The Proposed Fusion Algorithm

**FIGURE 1:**
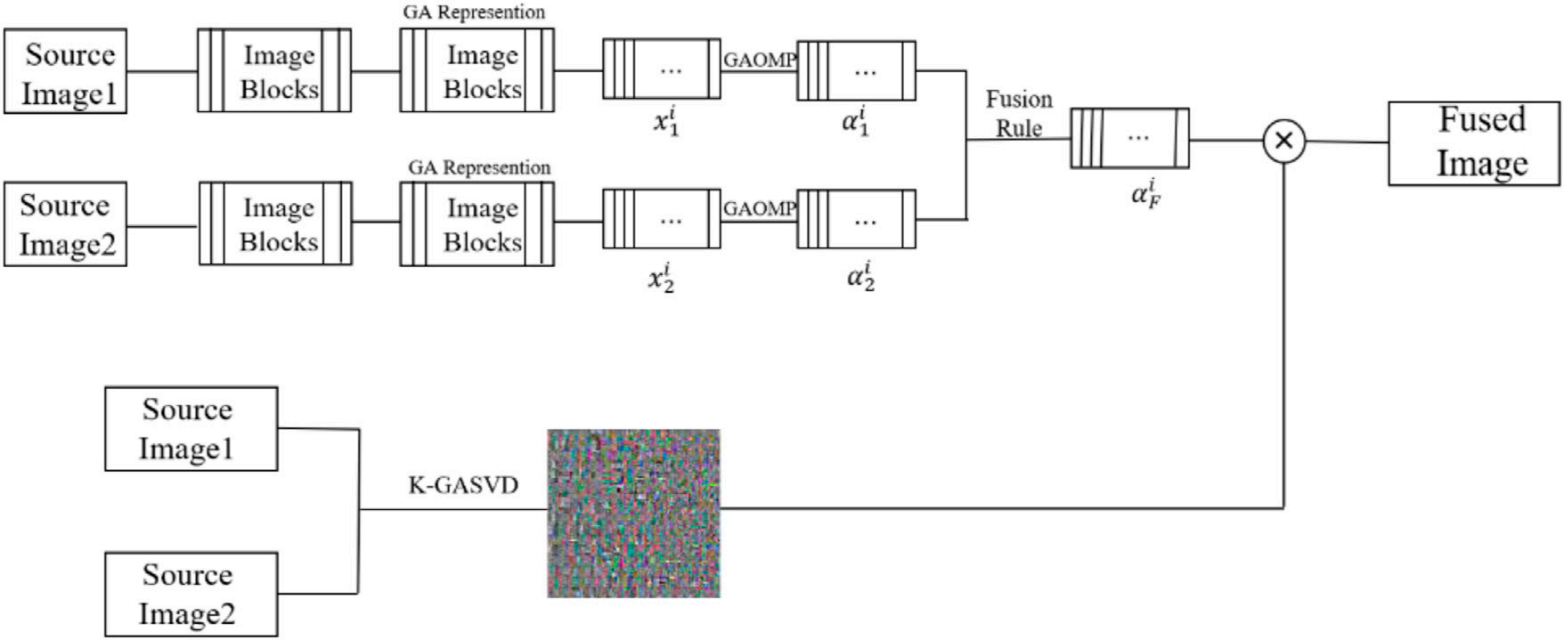
The framework of GA-SR based multi-modal medical image fusion.

Let $M_{1}$ and $M_{2}$ represent two multi-modal medical source images, respectively, and the framework of GA-SR based multi-modal medical image fusion is shown in Figure 1.

(1) The sliding window technique is introduced to divide the two source images into several sub-image blocks. The size of the sliding window is generally n×n and the step size is 1. The image blocks are converted into column vectors, and the i th image block is formed into the column vector which can be denoted as $x_{1}^{i}$ , $x_{2}^{i}$ .
(2) The sparse representation coefficients $\alpha_{1}^{i}$ and $\alpha_{2}^{i}$ of the column vectors can be calculated by GAOMP algorithm respectively in Wang et al. (2019b), which are described as follows

$$\alpha_{1}^{i}=argmin_{1}{\left\|x_{1}^{i}-DA\right\|}_{2}^{2},\;\;s.t.\left\|\alpha_{1}^{i}\right\|\leq J$$ (15)

$$\;\alpha_{2}^{i}=argmin_{2}\left\|x_{2}^{i}-DA_{2}^{2}\right\|,\;\;\;s.t.\left\|\alpha_{2}^{i}\right\|\leq J$$ (16)

where *D* represents the adaptive dictionary of image blocks obtained by dictionary training, $\alpha_{1}^{i}$ and $\alpha_{2}^{i}$ respectively represent the sparse coefficient vectors obtained by GAOMP, which can be combined to obtain a sparse coefficient matrix. $\left\|\alpha^{i}\right\|\leq J$ is the cutoff condition for dictionary training.

(3) The fused sparse coefficient matrix is obtained by the L1 norm (Yanan et al., 2020) maximum rule. The L1 norm refers to the sum of the absolute values of the elements, and the L1 norm is the optimal convex approximation of the L0 norm, which is more efficient than the L0 norm and is easier to optimize the solution. The L1 coefficient of the corresponding columns of the two sparse coefficient matrices are calculated, and the column with the larger norm is used as the column of the fused sparse coefficient matrix. The fusion rules of the sparse coefficients are as Eq. 17

$$\alpha_{F}^{i}=\{\begin{array}{c} \alpha_{1}^{i},\;\;{\left\|\alpha_{1}^{i}\right\|}_{1}\geq {\left\|\alpha_{2}^{i}\right\|}_{1}\\ \alpha_{2}^{i},\;\;{\left\|\alpha_{1}^{i}\right\|}_{1}<{\left\|\alpha_{2}^{i}\right\|}_{1}\; \end{array}$$ (17)

(4) A dictionary training algorithm is used to obtain the dictionary required for sparse representation. K-SVD is a classic dictionary training algorithm (Fu et al., 2019) in sparse representation. The K-GASVD algorithm in (Wang et al., 2019b) consists of two steps, which are sparse coding (Sandhya et al., 2021) and dictionary update (Thanthrige et al., 2020). The K-GASVD algorithm is used to perform and update dictionary training on the obtained sparse coefficient matrix.
(5) The fusion result of $x_{1}^{i}$ and $x_{2}^{i}$ can be obtained according to the GA-SR model of the three-channel multi-modal medical image, as shown in the Eq. 18

$$x_{F}^{i}=D\alpha_{F}^{i}$$ (18)

(6) All image patches are processed in the same way, the image block vector $x_{F}^{i}$ is calculated and converted into data sub-blocks. Finally, we can obtain a new image block and compose the final fused image $Q_{F}$ .

## 4 Experimental Analysis

**FIGURE 2:**
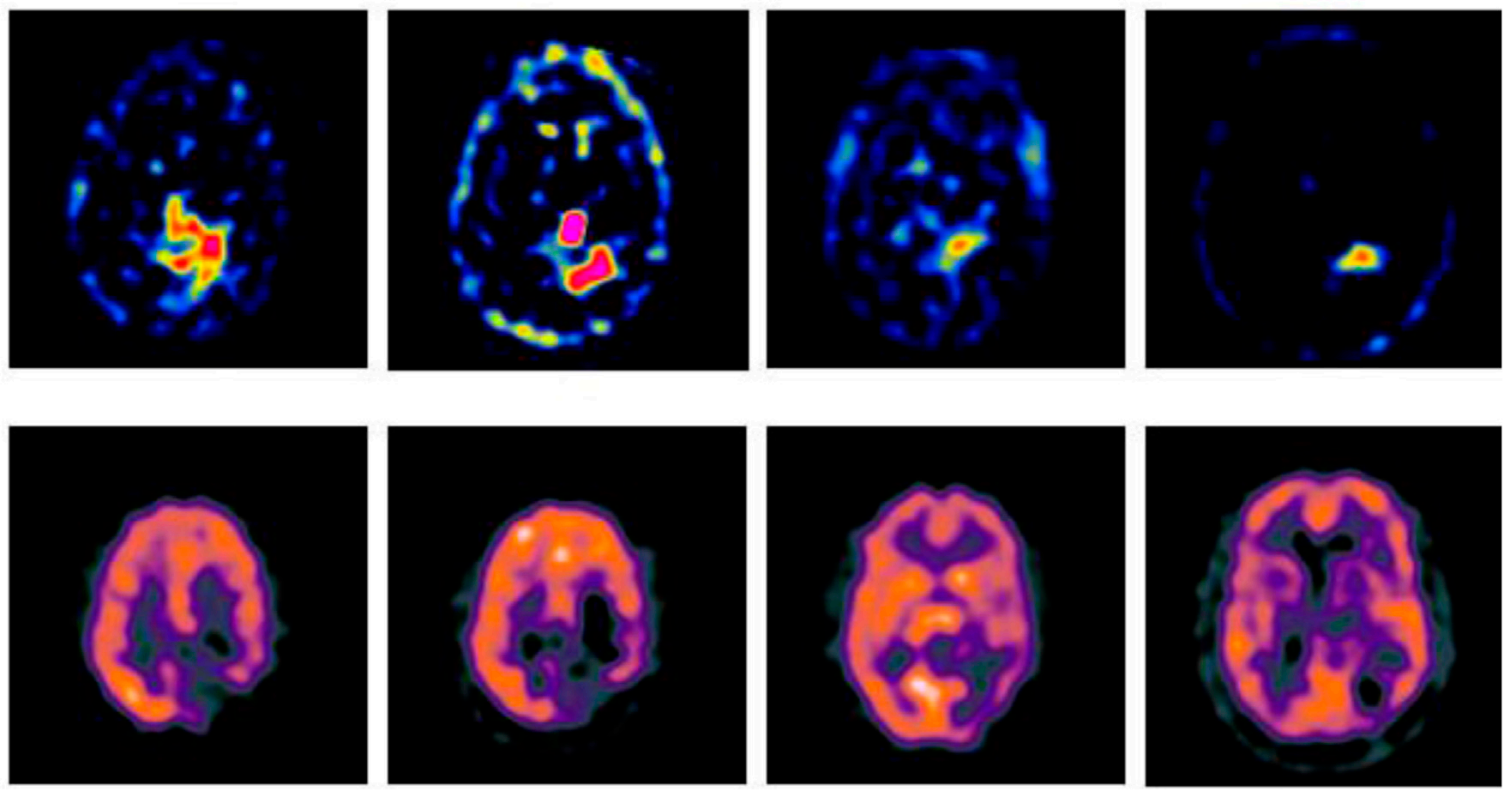
Source images.

In order to verify the effectiveness of the GA-SR based multi-modal medical image fusion, the experiments are implemented on four groups of multi-modal medical images selected from Harvard Medical School Database in Matlab with other exiting methods, such as Laplacian Pyramid algorithm (Liu et al., 2019), DWT-DBSS algorithm (Guruprasad et al., 2013), SIDWT-Haar algorithm (Xin et al., 2013) and Morphological Difference Pyramid algorithm (Matsopoulos et al., 1995). The source images are SPECT images obtained with different radionuclide elements, respectively. The spatial resolution of each image is 256 × 256. The source images used in the experiments are shown in Figure 2.

### 4.1 Subjective Quality Evaluation

**FIGURE 3:**
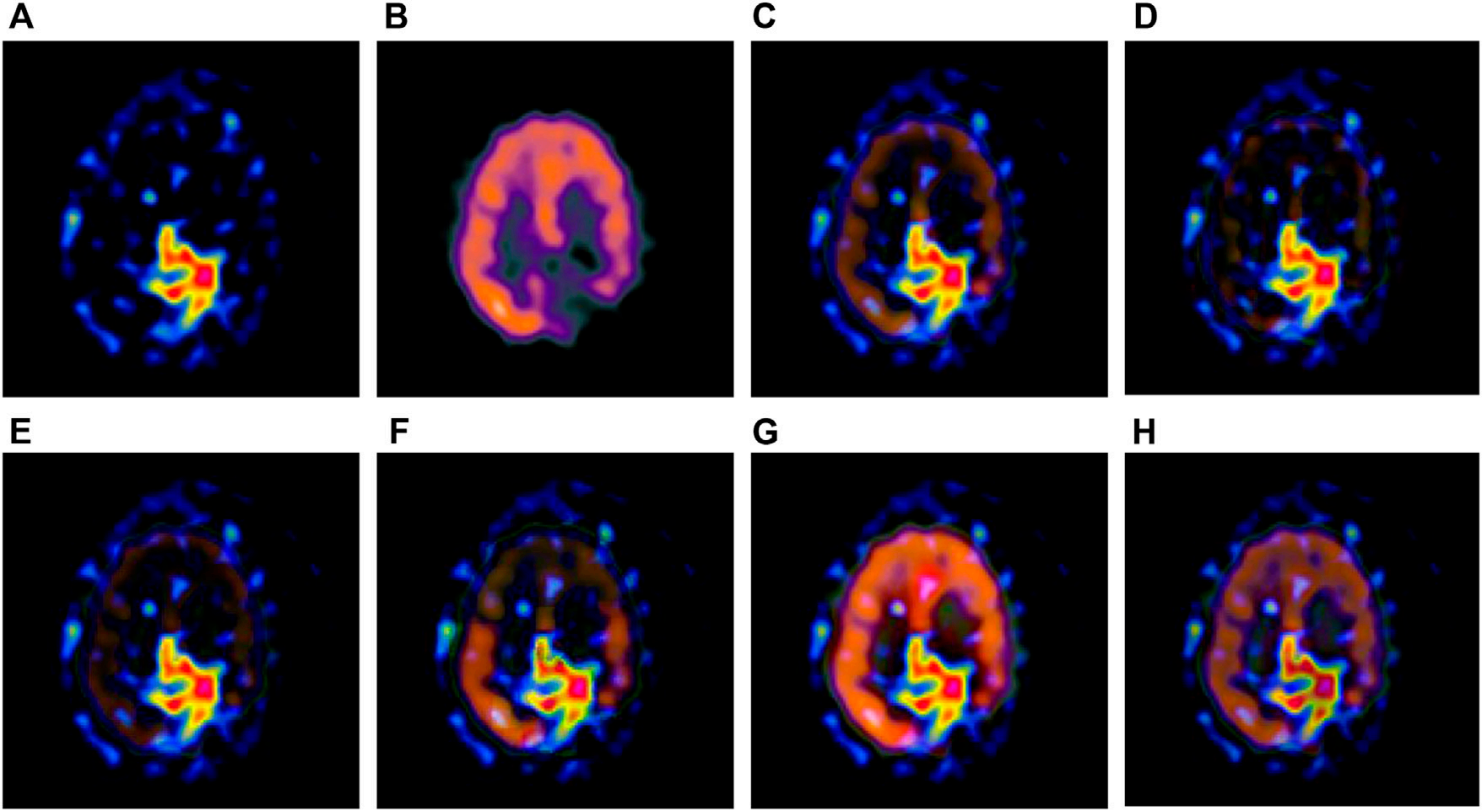
Fusion results of the first group. **(A)** Source image SPECT-TL1 **(B)** Source image SPECT-TC1 **(C)** Laplacian Pyramid **(D)** DWT-DBSS **(E)** SIDWT-Haar **(F)** Morphological Difference Pyramid **(G)** SR **(H)** GA-SR.

**FIGURE 4:**
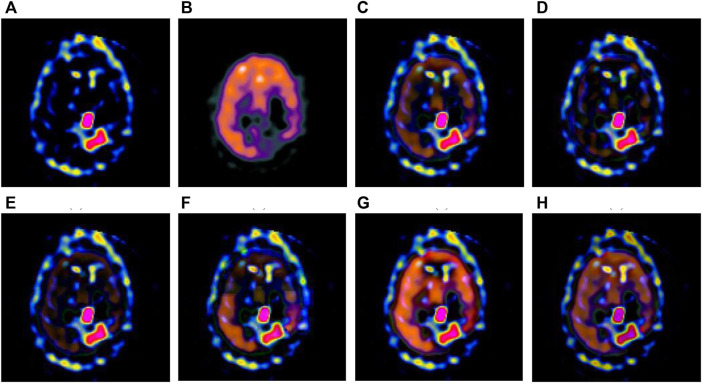
Fusion results of the second group. **(A)** Source image SPECT-TL2 **(B)** Source image SPECT-TC2 **(C)** Laplacian Pyramid **(D)** DWT-DBSS **(E)** SIDWT-Haar **(F)** Morphological Difference Pyramid **(G)** SR **(H)** GA-SR.

**FIGURE 5:**
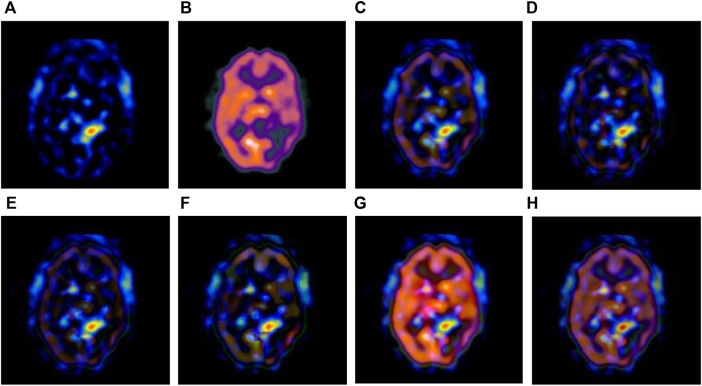
Fusion results of the third group. **(A)** Source image SPECT-TL3 **(B)** Source image SPECT-TC3 **(C)** Laplacian Pyramid **(D)** DWT-DBSS **(E)** SIDWT-Haar **(F)** Morphological Difference Pyramid **(G)** SR **(H)** GA-SR.

**FIGURE 6:**
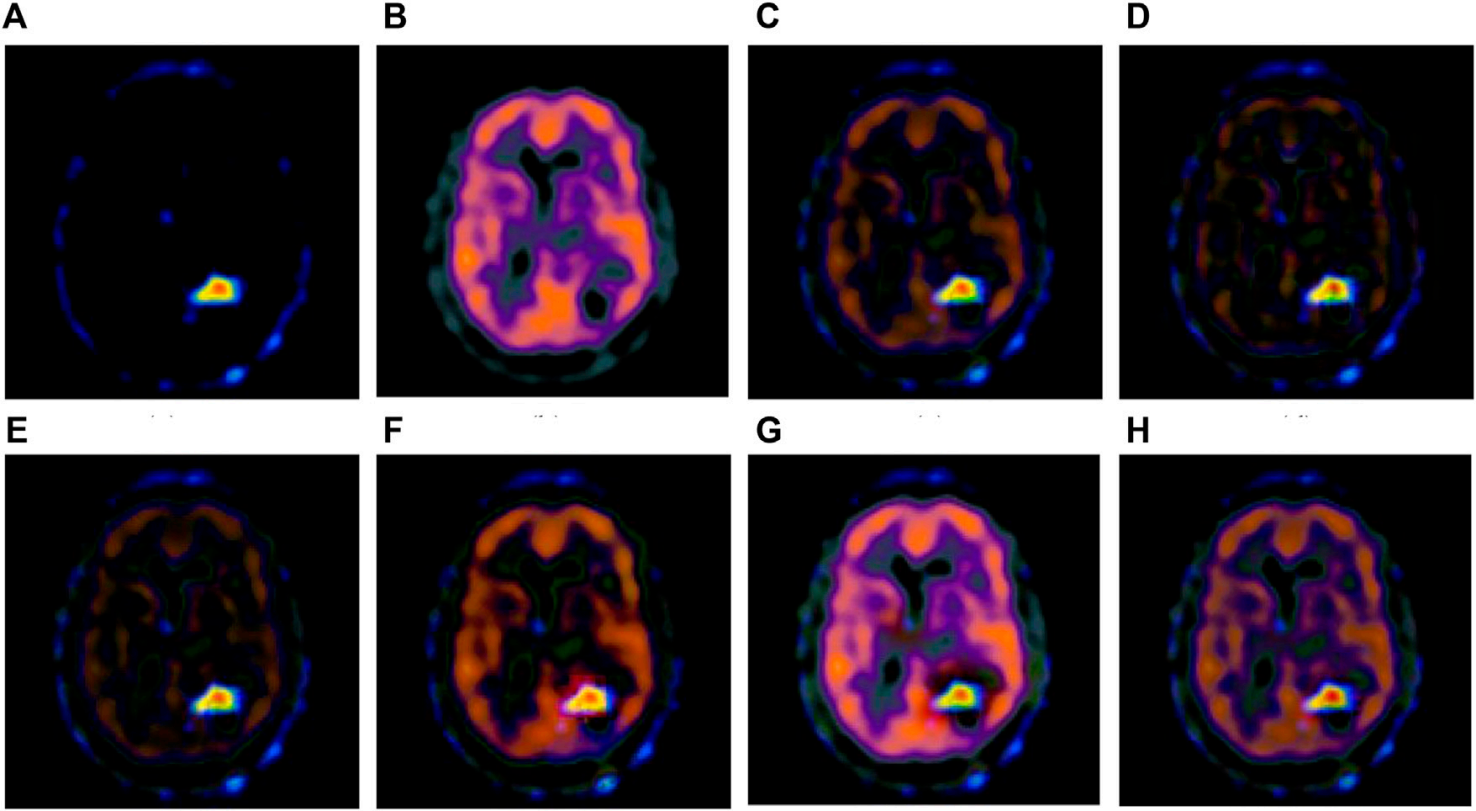
Fusion results of the fourth group. **(A)** Source image SPECT-TL4 **(B)** Source image SPECT-TC4 **(C)** Laplacian Pyramid **(D)** DWT-DBSS **(E)** SIDWT-Haar **(F)** Morphological Difference Pyramid **(G)** SR **(H)** GA-SR.

The multi-modal medical images are fused by six different algorithms respectively, and the obtained results are shown in Figures 3–6.

Figures 6A,B in each group are the source images used in the experiment, and Figures 6C–H are the fused results obtained by the six different algorithms. Subjectively, it can be seen that the edge of the images obtained by the first four algorithms is relatively complete, but the middle part is darker. The contrast and clarity of the images are low, which indicates that these four algorithms cannot fuse the two source images completely. As a result, the fused image information is incomplete. It can be seen that the fused images obtained by the SR algorithm and GA-SR algorithm are relatively complete, which can comprehensively cover the color and structure information of the two source images, and the fused images obtained are relatively clear. However, there are multiple red spots of different sizes in the images obtained by the SR algorithm, which cause the result to be distorted. The red spots will cover the correct information of the source image, which is not conducive to clinical diagnosis. As can be seen from each group of Figure 6H, the images are relatively clear, and there is no obvious occlusion area. The contrast of the images is relatively high, which indicates that the fused images obtained by the GA-SR algorithm can comprehensively cover the source image. It can provide comprehensive pathological information for medical staff and convenience for clinical medicine.

### 4.2 Objective Quality Evaluation

The evaluation indicators are adopted for objective evaluation of image quality. In this paper, four indicators of CC (Correlation Coefficient) (Li and Dai, 2009), PSNR (Peak Signal to Noise Ratio) (Hore and Ziou, 2010), RMSE (Root Mean Square Error) (Zhao et al., 2020) and Joint-Entropy (Okarma and Fastowicz, 2020) are used for performance analysis with the six fusion algorithms, and four groups of tables are obtained respectively, as shown in Tables 1–4.

**TABLE 1:** Quality evaluation of fused images of the first group.

Evaluation standard	Laplacian pyramid	DWT-DBSS	SIDWT-Haar	Morphological difference pyramid	SR	GA-SR
CC	0.6923	0.6284	0.6481	0.6842	0.7058	**0.74135**
Joint-Entropy	3.4538	3.4572	3.3152	3.4986	5.8191	**5.9109**
PSNR	17.442	17.5662	17.7478	17.0234	15.6494	**17.6253**
RMSE	0.1342	0.1323	0.7335	0.1409	0.1644	**0.1309**

Column 1 of the table is the Evaluation standard. The other columns of the table are the evaluated values of different methods.

**TABLE 2:** Quality evaluation of fused images of the second group.

Evaluation standard	Laplacian pyramid	DWT-DBSS	SIDWT-Haar	Morphological difference pyramid	SR	GA-SR
CC	0.6556	0.5840	0.602	0.6400	0.6832	**0.71145**
Joint-Entropy	3.9194	3.7748	3.7609	3.9974	**6.67605**	6.6487
PSNR	16.3629	16.5158	16.7125	15.8303	14.8449	**16.8667**
RMSE	0.1520	0.1494	0.1460	0.1616	0.1803	**0.1362**

Column 1 of the table is the Evaluation standard. The other columns of the table are the evaluated values of different methods.

**TABLE 3:** Quality evaluation of fused images of the third group.

Evaluation standard	Laplacian pyramid	DWT-DBSS	SIDWT-Haar	Morphological difference pyramid	SR	GA-SR
CC	0.7046	0.6472	0.6665	0.6737	0.6889	**0.7017**
Joint-Entropy	3.6714	3.7782	3.5535	3.5208	6.7404	**6.9520**
PSNR	16.8556	16.7812	17.0263	16.6019	15.194	**17.1890**
RMSE	0.1436	0.1449	0.1408	0.1479	0.1732	**0.1327**

Column 1 of the table is the Evaluation standard. The other columns of the table are the evaluated values of different methods.

**TABLE 4:** Quality evaluation of fused images of the fourth group.

Evaluation standard	Laplacian pyramid	DWT-DBSS	SIDWT-Haar	Morphological difference pyramid	SR	GA-SR
CC	0.6510	0.5897	0.6267	0.6235	0.56865	**0.6582**
Joint-Entropy	3.6212	3.6904	3.4017	3.9315	**7.0763**	6.61685
PSNR	17.0485	17.0083	17.1788	16.5814	15.0864	**17.2829**
RMSE	0.1405	0.1411	0.1384	0.1482	0.1754	**0.1362**

Column 1 of the table is the Evaluation standard. The other columns of the table are the evaluated values of different methods.

For fusion of the four groups, the CC of each group of images obtained by the GA-SR algorithm is higher than that obtained by other algorithms, indicating that the correlation of the images obtained by the GA-SR algorithm with the source image is higher, and the obtained image information is more complete. At the same time, the PSNR and RMSE of the images obtained by the GA-SR algorithm are higher than those obtained by other algorithms, indicating that the fused images obtained by the GA-SR algorithm are closer to the source images and have less distortion and more comprehensive information (Xiao et al., 2021; Gao et al., 2022a; Gao et al., 2022b; Gao et al., 2022c).

### 4.3 Further Analysis

**FIGURE 7:**
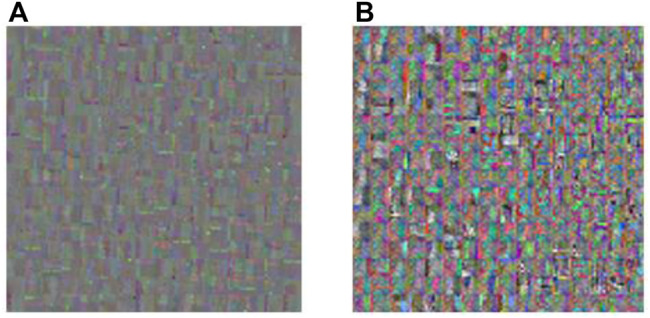
Dictionary obtained by K-SVD and K-GASVD. **(A)** Dictionary obtained by K-SVD algorithm **(B)** Dictionary obtained by K-GASVD algorithm.

Dictionary training is very important for sparse representation, and the quality of the dictionary directly affects the quality of image fusion. The dictionaries training based on the K-SVD and K-GASVD algorithms can be obtained respectively, as shown in Figure 7.

Figure 7A is the dictionary image obtained by the K-SVD algorithm, and Figure 7B is the dictionary image obtained by the K-GASVD algorithm. It is obvious that the color of the dictionary image obtained by the K-SVD algorithm is relatively single, that is because the K-SVD algorithm cannot fully handle the spectral components of the source image, resulting in the generated dictionary image containing a large number of gray image blocks. The dictionary image of K-GASVD contains richer comprehensive information.

**FIGURE 8:**
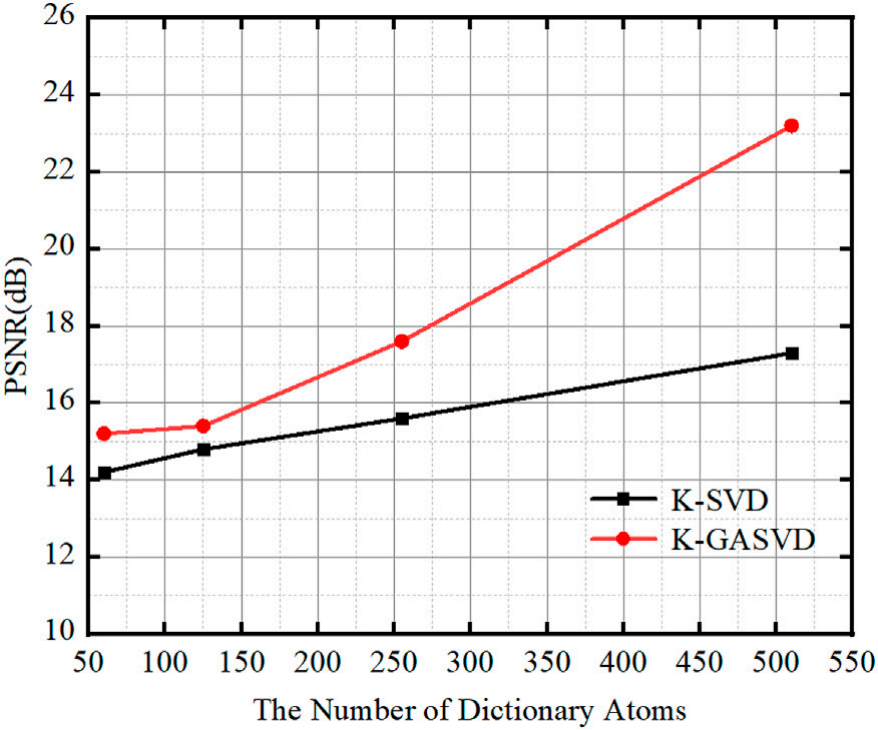
The relationship between the PSNR value of the image obtained based on K-SVD and K-GASVD and the number of dictionary atoms.

In order to verify the effect of the number of dictionary atoms on the quality of the fused image, we change the number of dictionary atoms to obtain different dictionary images, and finally obtain corresponding fused images. The relationship between the PSNR and the atomic number of fused images obtained from dictionaries with different atomic numbers is shown in Figure 8.

We can find that the PSNR of the fused images obtained by the K-GASVD model is significantly higher than that of the K-SVD with the increase of the number of dictionary atoms. On the other hand, the number of dictionary atoms required by the K-GASVD model is about 3/10 of the number of atoms required by the K-SVD model if the PSNR is same. Therefore, the number of atoms used in the K-GASVD is significantly reduced in the realization of the same fusion performance, which can present more colorful structures.

For computational complexity, it usually requires longer computational time for multi-modal medical image fusion than other existing real-valued algorithms, because of the non-commutativity of geometric multiplication. Inspired by the work in (Wang et al., 2021b), reduced geometric algebra (RGA) will be introduced to improve our algorithm with lower computational complexity.

## 5 Conclusion

In this paper, the multi-modal medical image is represented as a multi-vector, and the GA-SR model is introduced for multi-modal medical image fusion to avoid losing the correlation of channels. And the dictionary learning method based on geometric algebra is provided for more specific disease information for diagnosis. The experimental results validate its rationality and effectiveness. At next steps, we will focus on the analysis and diagnosis of pathological information using GA-SR based multi-modal medical image fusion.

## Data Availability

The original contributions presented in the study are included in the article/supplementary material, further inquiries can be directed to the corresponding author.
